# Epinephrine Production in Th17 Cells and Experimental Autoimmune Encephalitis

**DOI:** 10.3389/fimmu.2021.616583

**Published:** 2021-02-22

**Authors:** Pinguang Yang, Hong Tian, Yong-Rui Zou, Pierre Chambon, Hiroshi Ichinose, Gerard Honig, Betty Diamond, Sun Jung Kim

**Affiliations:** ^1^ Center of Autoimmune and Hematopoietic and Musculoskeletal diseases, The Feinstein Institute for Medical Research, Manhasset, NY, United States; ^2^ Donald and Barbara Zucker School of Medicine, Hofstra/Northwell, Hempstead, NY, United States; ^3^ Institute for Genetics and Cellular and Molecular Biology, Institute of Advanced Study of the University of Strasbourg, Strasbourg, France; ^4^ Department of Life Science, Graduate School of bioscience and Biotechnology, Tokyo Institute of Technology, Yokohama, Japan; ^5^ Crohn’s & Colitis Foundation, National Headquarters, New York, NY, United States

**Keywords:** epinephrine, blood–brain barrier, tyrosine hydroxylase, Th17, experimental autoimmune encephalitis

## Abstract

Epinephrine is a hormone secreted primarily by medullary cells of the adrenal glands which regulates permeability of blood–brain barrier (BBB). Recent studies showed signaling by epinephrine/epinephrine receptor in T cells is involved in autoimmune diseases. Nevertheless, the production of epinephrine by T cells and its pathogenic function in T cells are not well investigated. Our results show that phenylethanol N-methyltransferase (PNMT), a rate-limiting enzyme of epinephrine synthesis, is specifically expressed *in vitro* in differentiated T_H_17 cells and in tissue-resident T_H_17 cells. Indeed, expression levels of enzymes involved in epinephrine production are higher in T_H_17 cells from animals after EAE induction. The induction of PNMT was not observed in other effector T cell subsets or regulatory T cells. Epinephrine producing T_H_17 cells exhibit co-expression of GM-CSF, suggesting they are pathogenic T_H_17 cells. To delineate the function of epinephrine-production in T_H_17 cells, we generated a T_H_17-specific knockout of tyrosine hydroxylase (Th) by breeding a Th-flox and a ROR-gt-CRE mouse (Th-CKO). Th-CKO mice are developmentally normal with an equivalent T lymphocyte number in peripheral lymphoid organs. Th-CKO mice also show an equivalent number of T_H_17 cells *in vivo* and following *in vitro* differentiation. To test whether epinephrine-producing T_H_17 cells are key for breaching the BBB, migration of T cells through mouse brain endothelial cells was investigated *in vitro*. Both epi+ wild-type and epi- T_H_17 cells migrate through an endothelial cell barrier. Mice were immunized with MOG peptide to induce experimental autoimmune encephalitis (EAE) and disease progression was monitored. Although there is a reduced infiltration of CD4+ T cells in Th-CKO mice, no difference in clinical score was observed between Th-CKO and wild-type control mice. Increased neutrophils were observed in the central nervous system of Th-CKO mice, suggesting an alternative pathway to EAE progression in the absence of T_H_17 derived epinephrine.

## Introduction

Experimental allergic encephalitis (EAE), a mouse model for autoimmune demyelination of the central nervous system (CNS), has been widely used to investigate pathogenic mechanisms for the development of human multiple sclerosis (MS). It is a CD4^+^ T cell-mediated disease with a major contribution from T-helper cell 17 (T_H_17) cells. T_H_17 cells are characterized by their expression of the transcription factor RORγt and production of the hallmark cytokines IL-17 and IL-22 (1, 2). TGFβ, in the presence of proinflammatory cytokines such as IL-6 and IL-21, drives the expression of RORγt and promotes T_H_17 differentiation, where TGFβ alone induces Foxp3 and promotes CD4^+^ T regulatory cell differentiation. Furthermore, IL-23 and inflammation-induced serum amyloid A1 promote the terminal differentiation of T_H_17 cells into mature pathogenic cells (3, 4). Stimulation with IL-23 induces expression of granulocyte-macrophage colony-stimulating factor (GM-CSF) in T_H_17 cells. GM-CSF plays an essential role in the encephalitogenicity of T cells in EAE. GM-CSF promotes microglial cell activation which is required for EAE induction (5). It also coordinates the recruitment of CCR2^+^Ly6C^high^ monocytes into the CNS and subsequently promotes tissue injury (6).

In EAE, pathogenic CD4 T cells are primed in peripheral lymph nodes and migrate into the CNS to initiate tissue inflammation. T_H_17 cells are among the first infiltrating blood leukocytes, however, it is unclear how they gain access to the CNS to initiate disease. CCR6, a chemokine receptor specific for T_H_17 cells, is required for the initial insult. CCR6^+^ T_H_17 cells enter the CNS through the choroid plexus where epithelial cells constitutively express CCL20, the ligand for CCR6 (7). T_H_17 cells disseminate *via* the CSF into the subarachnoid space of the CNS. There, T_H_17 cells are locally reactivated and induce an inflammatory environment which triggers the recruitment of a second wave of pathogenic cells through blood–brain barrier (BBB) (8). However, the mechanism of BBB breach which allows leukocyte infiltration is not clearly understood. There are several molecules which can breach BBB integrity (9). Our group previously found that epinephrine levels in blood regulate permeability of the BBB, but there is a report also epinephrine protect BBB integrity (10, 11); thus, a careful analysis of epinephrine-producing cells and their ability to infiltrating the CNS is warranted.

In this study, we investigated whether epinephrine is produced by T_H_17 cells and whether epinephrine contributes to BBB breach and EAE pathogenesis. We found that epinephrine is selectively produced in T_H_17 cells, and knockdown of tyrosine hydroxylase (Th) inhibits epinephrine expression in T_H_17 cells. We hypothesized that epinephrine expression in Th17 cells is required for EAE induction. There was a decreased T cell infiltration but increased neutrophil infiltration into the CNS of Th deficient mice, but there was no difference in kinetics of disease development or severity in the presence or absence of epinephrine producing T_H_17 cells, suggesting multiple mechanisms of disease pathogenesis and BBB breach.

## Materials and Methods

### Mice

*Th* CKO mice were generated by breeding of *RORc*-*Cre* (stock no: 022791, purchased from Jackson Laboratory, Bar Harbor, ME) and Th flox mice [kindly provided by Dr. Ichinose and Champbon (12, 13)]. *Th*-CKO mice (*Th^fl/fl^ RORc*-*Cre*+) and littermate control mice (*Th^fl/fl^ RORc*-*Cre*-) were maintained in a specific pathogen-free facility at the Feinstein Institutes for Medical Research (FIMR). Wild type C57BL/6 mice and FOXP3-GFP (stock number 023800) were purchased from Jackson Laboratory. All the experiments strictly followed the guideline in the Guide for the Care and Use of Laboratory Animals of the National Institutes of Health, and the protocol was approved by the Committee on the Ethics of Animal Welfare of the FIMR (protocol number 2009-048).

Sample size and number of experiments to achieve adequate power was chosen based on previous studies with similar methods. We randomized the mice from different cages and different time points to exclude batch variation.

### Isolation of Human Tregs and T_H_17 Cells

Leukopacks were purchased from NY Blood Center (Long Island City, NY) and total PBMCs were isolated by Ficoll-Paque (Millipore Sigma, Burlington, MA) gradient centrifugation. Briefly, the leukopack was diluted with Hanks balanced saline solution (HBSS, Life Technologies) 1:2 (vol:vol) and layered on the Ficoll. Cells were centrifuged at 2,000 rpm for 20 min without break at room temperature (r.t.). PBMCs were collected from the intermediate layer and washed three times with HBSS.

To isolated CD4+ Treg cells and T_H_17 cells, PBMCs were stained with antibodies and live CD127-Treg cells (FVD-CD4+ CD127loCD49d-CD25+) (14), CD25+ Treg cells (FVD-CD4+CD25+), and TH17 cells (FVD-CD4+CD25-CCR6+CXCR3-) were isolated by FACSAria (BD Biosciences).

### T cell Differentiation *In Vitro*

Naive CD4+ T cells were isolated from the spleen using an Easysep naïve CD4+ enrichment kit (Stem cell technologies, Cambridge, MA). 10^6^ CD4+ T cells were incubated in a CD3e (145-2C11; BD Biosciences) (5 μg/ml) pre-coated plate with anti-CD28 (37.51; BD Biosciences) (5 μg/ml) alone or with differentiation cocktails for 4 days: for T_H_1 (10 μg/ml anti-IL-4 (11B11; BD Biosciences), 20 ng/ml IFNγ and 5 ng/ml IL-12), for T_H_2 (10 μg/ml anti-IFNγ (XMG1.2; BD Biosciences), 5 ng/ml IL-2, and 20 ng/ml IL-4), for T_H_17 (10 μg/ml of each neutralizing antibodies against IFNγ, IL-4, IL-2, and 8 ng/ml IL-6, 2 ng/ml TGFβ). All the antibodies were purchased form BD Biosciences (Franklin Lakes, NJ) and recombinant cytokines were purchased from Peprotech (Rocky Hill, NJ). Successful differentiation was confirmed by intracellular staining for cytokines after 6 h of PMA/ionomycine/BFA stimulation at the end of differentiation, including IFNγ (for T_H_1), IL-4 (for T_H_2), and IL-17 (for T_H_17).

### Isolation of IL-17+ CD4 T Cells

IL-17 producing CD4+ T cells were isolated using a mouse IL-17 secretion assay kit (Miltenyi Biotec) according to the manufacturer’s protocol with modifications. Total LNs or mononuclear cells from spinal cord (SC) were stimulated with PMA (10 µg/ml) and ionomycin (1 µg/ml) for 3 h and IL-17 was captured on the surface. Cells were stained with anti-IL-17 reagent (provided in the kit), FVD, TCRβ and CD4 antibodies to detect CD4+ T cells. After labeling, IL-17+ and IL-17-CD4+ T cells were isolated by FACAria.

### Antibodies and Reagents

Antibodies for western blotting, polyclonal anti-PNMT antibodies (ab90862), anti-ACTIN (ab8226), goat anti-rabbit IgG (HRP) (ab205718), and goat anti-mouse IgG (HRP) (ab205719), were purchased from Abcam (Cambridge, MA).

For flow cytometry, antibodies were purchased from eBioscience [anti-mouse CD4 (GK1.5), anti-mouse MHCII (M5/114.15.2), anti-mouse Ly6G (RB6-8C5), anti-mouse CD45 (30-F11), anti-mouse IFNγ (XMG1.2), anti-mouse IL17A (eBio17B7), anti-mouse FOXP3 (FJK-16s), anti-human CD127 (eBioRDR5), anti-human CD25 (BC9.6)], BioLegend [anti-mouse TCRβ (H57-597), anti-mouse CD11b (M1/70), anti-mouse CD8 (53-6.7), anti-human CD3e (SK7), anti-human CD4 (RPA-T4), anti-human CD49d (9F10), anti-human CCR6 (G034E3), ant-CXCR3 (G025H7)], or R&D systems [anti-mouse CCR6 (FAB5909)].

### qPCR and Semi-qPCR

Total RNA was purified from cells by using Direct zol RNA microprep (Zymo research, Irvine, CA), and quantity and quality of RNA was measured by bioanalyzer. Two hundred nanogram of total RNA was used for cDNA synthesis by iScript cDNA synthesis kit (Bio-rad), and qPCR was performed by Taqman primers with light cycler 2x master mix (Invitrogen) on Light cycler (Roche). Relative expression quantification was calculated using the ΔΔCt method with the Sequence Detection Systems Software, version 1.9.1 (Applied Biosystems) and level was normalized by *Polr2a*. Taqman primer were purchased from Invitrogen: Mm00839502_m1 (*Polr2a*), Mm00447557_m1 (*Th*), Mm01268876_m1 (*Th*), Mm00516688_m1 (*Ddc*), Mm00460472_m1 (*Dbh*), Mm00476993_m1 (*Pnmt*), Mm01309416_m1 (*Il7r*), Mm01290062_m1 (*Csf2*), Mm00439619_m1 (*Il17a*), Hs00165941_m1 (*TH*), Hs00160228_m1 (*PNMT*), Hs00172187_m1 (*POLR2A*).

Human *TH* expression was also quantified by semi-quantitative PCR, followed by primers and the condition for PCR was adopted from the previous study (15). Primers: 5′-TGTCAGAGCTGGACAAGTGT-3′ (TH-F) and 5′-GATATTGTCTTCCCGGTAGC-3′ (TH-R), 5′-AGACAGCAACTCTTCTCTGC-3′ (VMAT-F), 5′-CTATCCCTTGCAAGCAGTTGT-3′ (VMAT-R).

### *In Vitro* Transmigration Assay

*In vitro* T cell migration assay was set up following a published study (16). Primary mouse brain endothelial cells were purchased (Cell Biologics) and cultured with endothelial cell culture medium (supplemented with 10% FBS, P/S, glutamine, vascular endothelial growth factor, epidermal growth factor, endothelial cell growth supplement, hydrocortisone, and heparin). 10^5^ endothelial cells were seeded on the bottom of gelatin (Cell Biologics) and laminin (Becton Dickinson)-coated transwell filter inserts (6.5 mm diameter, 0.33 cm^2^ surface with 3.0 μm pore size) (Fluoro Block, Falcone) and culture until the endothelial cell layer become confluent (no leak the solution on the upper chamber). When the endothelial cell layer is ready for assay, IL-17+ T_H_17 cells or IL-17- non-T_H_17 cells were isolated from EAE induced mice and labeled with CFSE (15 μM) (Molecular Probe, Life Technologies). Loaded in the upper chamber were 2.5 x 10^5^ cells. The recombinant chemokine, CCL20 (50 ng/ml) (R&D Systems) was added in the lower chamber. After 6 or 16 h at 37 °C in 5% CO2, migrated T cells were collected by centrifugation of the lower chamber medium and cell number was counted by fluorescence counter, Cellometer Auto2000 (Nexcelom).

T cells on the endothelial cell layer were fixed with 4% formaldehyde and stained with rabbit polyclonal anti-Claudin 5 (REF# 34-1600, Invitrogen) and anti-rabbit AF594 (Invitrogen). The membrane was removed from the transwell and transferred to a glass slide. Images were captured by Zeiss Apotome (20x magnification). Independent areas per sample were captured and number of T cells was counted.

### Induction of EAE

For active EAE induction, female mice were immunized subcutaneously at two sites in the posterior right and left flank with 200 ug MOG_35–55_ emulsified in CFA (Hooke Laboratories, made with 2 mg/mL heat-killed mycobacterium tuberculosis H37Ra/mL emulsion in complete Freund’s adjuvant). Pertussis toxin (PT) (List Biological Laboratories, 300 ng) was injected intraperitoneally on days 0 and 2 after immunization. Mice were observed for signs of EAE beginning on day 7 after immunization. Mice were clinically assessed with daily assignment of scores on a standard 0–5 scale as follows: no clinical expression of disease, 0; partially limp tail, 0.5; completely limp tail, 1; limp tail and waddling gait, 1.5; paralysis of one hind limb, 2; paralysis of one hind limb and partial paralysis of the other hind limb, 2.5; complete paralysis of both hind limbs, 3; ascending paralysis, 3.5; paralysis of trunk, 4; moribund, 4.5; death, 5.

### CNS–Infiltrating Mononuclear Cell Isolation

EAE mice were killed, and spinal cords were removed after mice were perfused through the left cardiac ventricle with PBS. Spinal cords were flushed out with PBS by hydrostatic pressure, minced and digested with 2 mg/mL collagenase D (Sigma) and 40 µg/mL DNAse I (Sigma-Aldrich) in HBSS at 37°C for 30–45 min. Following centrifugation, cells were resuspended in 4 mL of 30% Percoll (GE Healthcare) in HBSS, and 4 mL of 70% Percoll was underlayed in a 15 mL conical tube. Percoll gradient separation was performed by centrifugation at 1,576 rpm for 30 min at room temperature. Infiltrating mononuclear cells were collected at the interface layer of the Percoll gradient. Cells were washed and counted before analysis. Cells were labelled with antibody conjugated to fluorochrome dyes and analyzed by flow cytometry.

### Western Blotting

Cells were washed with ice-cold PBS and lysed in a radio immunoprecipitation assay buffer (RIPA buffer: 25 mM Tris-HCl [pH 7.6], 150 mM NaCl, 1% NP-40, 1% sodium deoxycholate, 0.1% SDS; Pierce, Rockford) with a complete protease inhibitor cocktail (Roche) and a phosphatase inhibitor cocktail (Sigma-Aldrich, Saint Louis, MO) for 30 min on ice. Total protein lysates were obtained from the supernatant after centrifugation and the protein concentration was measured using BCA protein assay kit (Pierce, Thermo Scientific). The lysates were separated by 4–12% Bis-Tris PAGE and transferred to PVDF membrane (Hybond-C; GE Amersham, UK). The membrane was blocked for 1 h at room temperature with 5% non-fat dry milk in 0.1% Tween-20 in Tris buffered saline (TBS-T) (pH 7.6) buffer. The blocked membranes were then incubated with primary antibodies overnight at 4 °C. The membrane was washed four times (15 min each) with TBS-T buffer, and then incubated with secondary antibodies conjugated with HRP (1:20,000) for 1h at room temperature. The immune-reactive proteins were visualized with ECL detection reagents (Thermo). The image was scanned, and the intensity of band was quantified by Li-COR (LI-COR Biosciences, NE).

### Epinephrine Measurement

Until analysis, 1x10^6^ Th0 or Th17 cells were washed with ice-cold PBS, snap-frozen and kept at −80 °C. Epinephrine and norepinephrine were quantified by LC-MS from the mass spectrometry facility at Cold Spring Harbor Laboratory.

### Statistics

Statistical significance was calculated and determined by a nonparametric, Mann-Whitney test, and p ≤ 0.05 was considered significant. No sample was excluded.

## Results

### Selective Expression of Epinephrine in *In Vitro* Differentiated T_H_17 Cells

**Figure 1:**
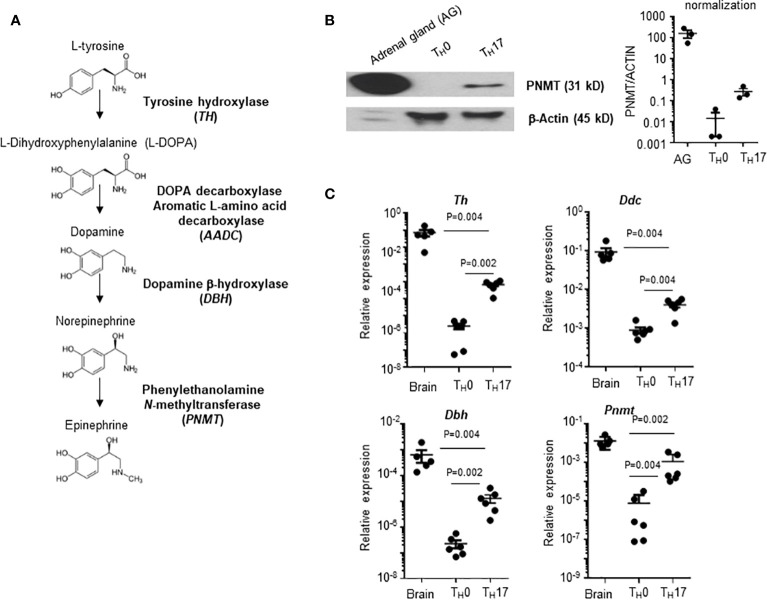
PNMT expression in T_H_17 cells. **(A)** Diagram of *de novo* epinephrine synthesis and converting enzymes. **(B)** PNMT expression in T_H_17 cells. Naïve CD4+ T cells from spleens were isolated and T_H_0 or T_H_17 cells were differentiated. Lysates of each cell subset (20 μg each) and adrenal gland tissue lysates (1 μg) were used for western blotting. Expression of actin was detected as a control protein. One representative image from 3 independent experiments. Graph is a quantitation and normalization of PNMT to ACTIN. **(C)** Total RNA was prepared and levels of *Th*, *Ddc*, *Dbh*, and *Pnmt* mRNA was measured by qPCR. RNA samples from the mouse brain (control mice) were used as positive controls. Relative expression was normalized to the level of Actin. Each dot represents an individual sample and the bar represent mean ± SEM (n = 5).

Infiltration of pathogenic T cells into the CNS is a critical step of EAE pathogenesis, but the mechanism of BBB breach is not clearly understood. Epinephrine is a stress molecule which can cause a breach of BBB integrity (10). Therefore, we measured whether the epinephrine synthesis pathway is induced in T_H_ cells. There are four enzymes (Tyrosine hydroxylase (*TH*), DOPA decarboxylase aromatic L-amino acid decarboxylase (*AADC*), dopamine b-hydroxylase (*DBH*) and pheynylethanol N-methyltransferase (*PNMT*)) involved in epinephrine synthesis (**Figure 1A**), and PNMT is the rate-limiting enzyme (17). We induced mouse CD4 T_H_ cell subsets by *in vitro* differentiation methods (18), and PNMT expression was measured. PNMT expression was strong in adrenal gland tissue, which is a positive control for the assay, and was detected in Th17 cells (**Figure 1B**). We also measured the expression of three other enzymes, *Th*, *Aadc*, and *Dbh*, involved in epinephrine synthesis in T_H_17 cells by qPCR, and all the enzymes were expressed (**Figure 1C**). Since all the enzymes are expressed in T_H_17 cells, we assessed epinephrine production. Epinephrine was detected in T_H_17 cell cultures and at much lower concentration in T_H_0 cells (**Table 1**), confirming secretion of epinephrine by T_H_17 cells.

**Table 1:** Epinephrine and norepinephrine level in T cells.

Sample	TH0	TH17	P-value
Norepinephrine	0.06 ± 0.02 (ng/mg)	0.36 ± 0.16 (ng/mg)	0.068
Epinephrine	0.01 ± 0.01 (ng/mg)	0.03 ± 0.01 (mg/mg)	0.026

**Figure 2:**
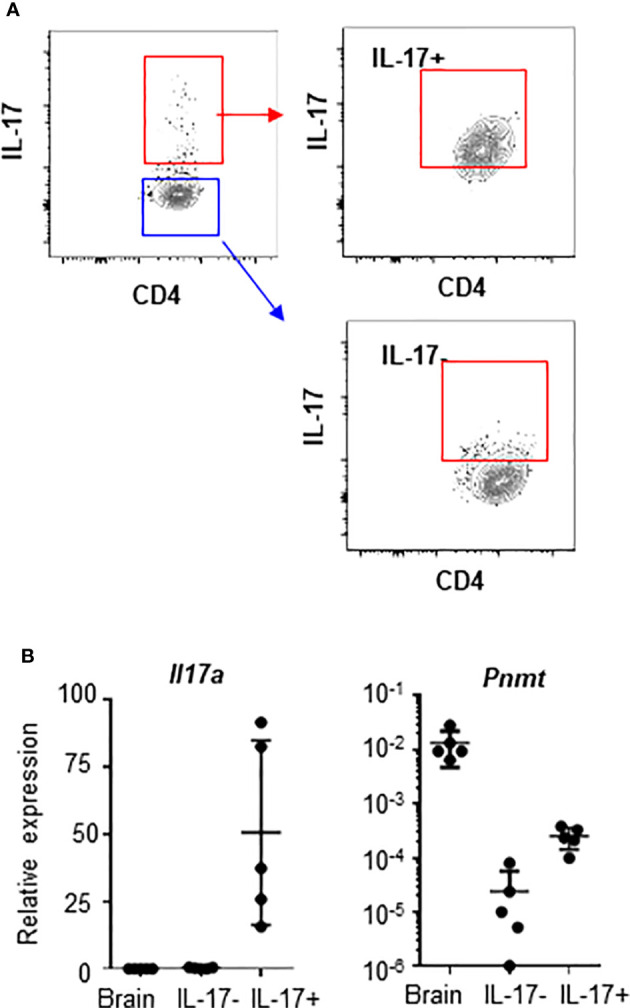
*Pnmt* expression in T_H_17 cells in dLNs. CD4+ T cells were isolated from LNs from 6-8 weeks old C57BL/6 mice. **(A)** IL-17+ T_H_17 cells and IL-17- non-T_H_17 cells were enriched and isolated by cell sorter. Purity of IL-17+ and IL-17- cells after sorting was confirmed and ~75% of cells are enriched in the positive fraction and less than 5% of cells are contaminated in the negative fraction. **(B)** Total RNA was prepared from the sorted cells and the levels of *Il17A* and *Pnmt* mRNA was measured by qPCR. RNA from whole brain tissue was used as a positive control. Relative expression was calculated based on the level of *Polr2a*. Each dot represents an individual mouse and the bar represents mean ± SEM (n = 5).

We also questioned whether *in vivo* generated T_H_17 cells express genes involved in epinephrine synthesis. We isolated T_H_17 cells and non-T_H_17 cells by their expression of IL-17 from LNs (**Figure 2A**). Mouse brain tissue was used as a positive control and *Il17a* and *Pnmt* expression was measured by qPCR. Expression of *Il17a* and *Pnmt* was detected only in T_H_17 cells but not in non-T_H_17 cells (**Figure 2B**).

**Figure 3:**
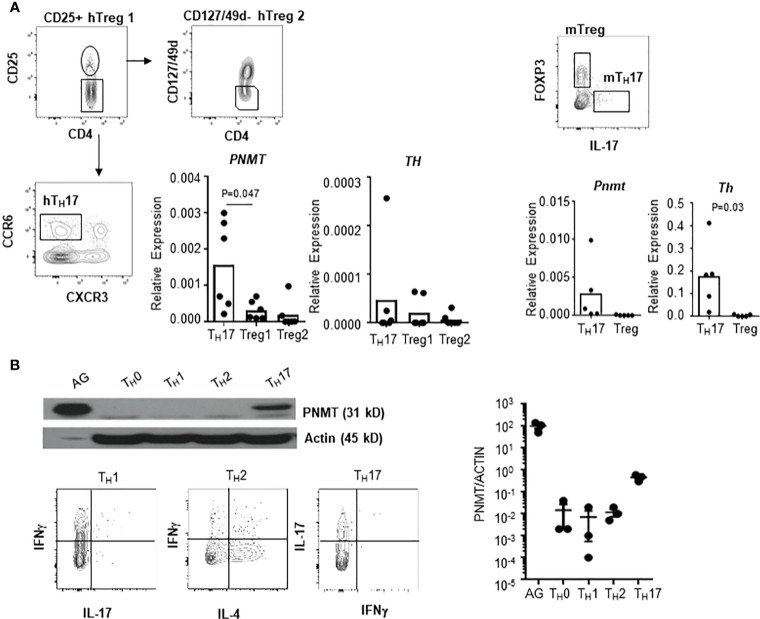
Th and PNMT expression in human and mouse helper T cell subsets. **(A)** *Th* and *PNMT* expression in Treg cells and Th17 cells. Human CD25+ Treg cells (hTreg1 in figure), CD127lo Treg cells (CD49d-CD127^lo^CD25+) (hTreg2 in figure) and T_H_17 cells (CD25-CXCR3-CCR6+) was isolated from PBMCs and murine FOXP3+ Treg and T_H_17 cells were isolated from spleen of 6-8 weeks of *Foxp3*-GFP C57BL/6 mice. Total RNA was isolated and *Th and PNMT* expression was measured by qPCR. One representative image from 2 independent experiments (n = 6). **(B)** Mouse CD4+ T_H_ cells were differentiated from naïve CD4+ T cells and protein level of PNMT was measured by western blot. Differentiation was confirmed by cytokine production of IFNγ (T_H_1), IL-4 (T_H_2), and IL-17 (T_H_17) at the end of culture. Levels of actin was measured as a protein loading control. One representative image of 3 independent experiments. Intensity of protein expression was quantified by densitometry and normalized to the level of ACTIN.

There is a report that human regulatory T cells (CD25+ Treg) produce epinephrine (15). To confirm the specificity of epinephrine production in T_H_17 cells, we measured expression of *PNMT* and *TH* in Treg cells and T_H_17 cells. We isolated CD25+ Treg, which were analyzed in the previous study, CD25+CD127-CD49^lo^ Treg, which is the functional Treg subset, or circulating T_H_17 cells (CD25-CCR6+CXCR3-) from human peripheral blood mononuclear cells (PBMCs) (14). We also isolated FOXP3+ Tregs cells and IL-17+ T_H_17 cells from the mouse spleen. *PNMT* and *TH* expression was measured by qPCR. Contrary to previous reports, we could not detect expression of *TH* in Tregs and human blood T_H_17 cells (**Figure 3A**), even using primers which were used in the previous study (data not shown). We could detect low but significant level of *PNMT* expression in T_H_17 cells, but not Tregs (**Figure 3A**). Similar to the result in Tregs, other helper T cell subsets (T_H_1 and T_H_2) did not express PNMT (**Figure 3B**).

These data suggest that enzymes which are involved in epinephrine production are selectively induced in CD4+ T_H_ cell subsets, especially T_H_17 both *in vitro* and *in vivo*.

### Pathogenic T_H_17 Cells Express *Th* and *Pnmt*

**Figure 4:**
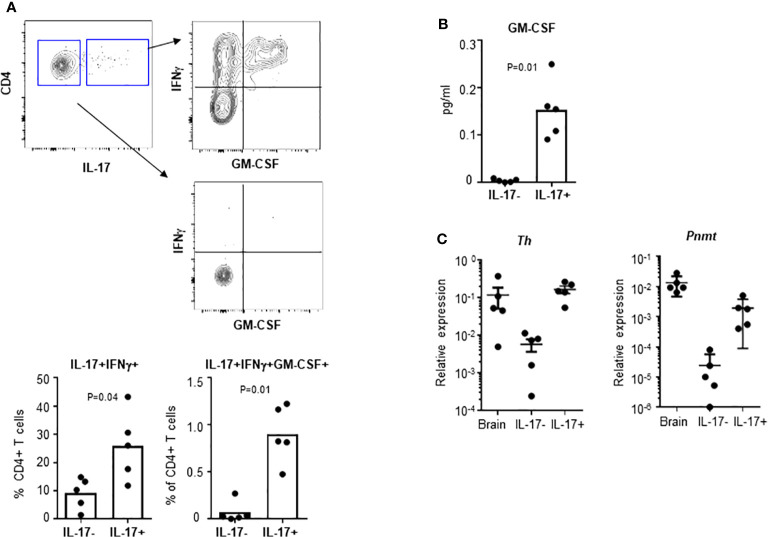
*Th* and *Pnmt* expression in pathogenic T_H_17 cells. **(A)** T_H_17 and non-T_H_17 cells were identified by IL-17 production and co-stained with IFNγ and GM-CSF in EAE induced animals. IFNγ- or IFNγ/GM-CSF-expressing pathogenic T_H_17 cells were quantified. IL-17+ T_H_17 and IL-17- non-T_H_17 cells were isolated and cultured overnight for GM-CSF in the supernatant **(B)**. Or the isolated cells were directly used for RNA isolation and *Th* and *Pnmt* was measured by qPCR **(C).** Each dot represents an individual animal and the bar represent the mean (n = 3).

Infiltrating pathogenic T_H_17 cells can be identified by co-expression of GM-CSF and sustained expression of IL7Ra (3, 19). To test whether pathogenic T_H_17 cells, which are induced during EAE, express genes involved in epinephrine production, we measured expression of *Th* and *Pnmt* in T_H_17 cells from EAE animals. DLNs were collected from day 10 EAE animals. IL-17+ T_H_17 cells and IL-17- non-T_H_17 cells were analyzed for expression of IFNγ and GM-CSF by flow cytometry. These cells were isolated and the expression of *Th* and *Pnmt* was measured by qPCR, too. As seen in **Figure 4A**, a high percentage of IL-17+ T_H_17 cells but not non-T_H_17 cells co-expressed IFNγ or IFNγ and GM-CSF. Secretion of GM-CSF was measured by ELISA (**Figure 4B**). These *in vivo* generated T_H_17 cells express both *Pnmt* and *Th* (**Figure 4C**), and interestingly, the level of expression of both gene is higher than in T_H_17 cells from non-immunized mice or *in vitro* differentiated T_H_17 cells (as in **Figure 1C**).

### *Th* Knockout Mice Have Normal Development of T_H_17 Cells Which Can Cross an Endothelial Barrier

**Figure 5:**
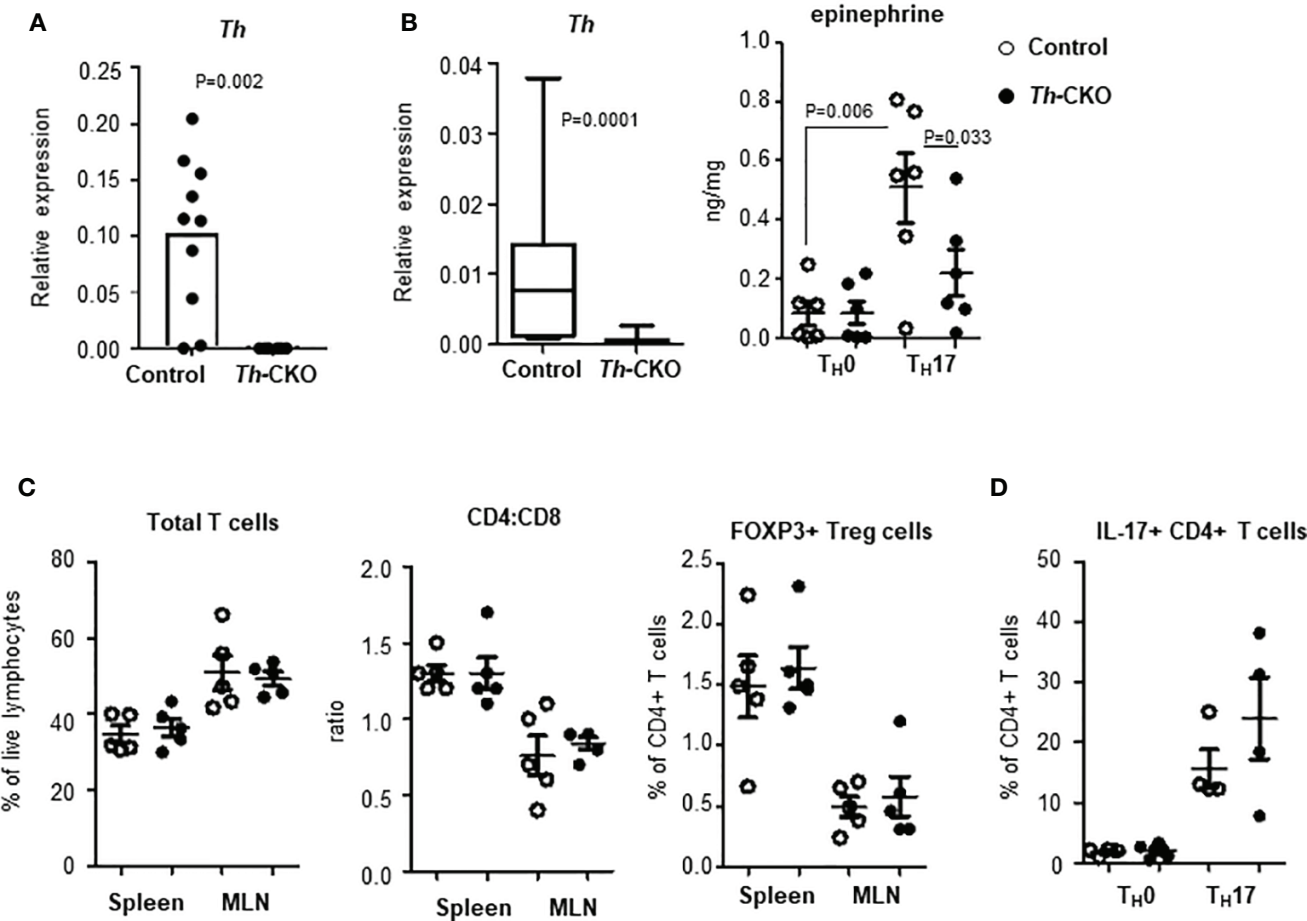
Epinephrine expression and lymphocytes in Th-CKO mice. **(A)** T_H_17 cells were isolated from SCs from control and Th-CKO EAE mice and *Th* expression was measured by qPCR. The relative expression was calculated to the level of *Porl2a*. Each dot represents an individual animal. 3 independent experiments (n = 6). **(B)** Naïve CD4+ T cells were isolated from spleens of 6-8 weeks old control and Th-CKO mice and differentiated into T_H_0 or T_H_17 cells. Total RNA was prepared and *Th* expression was measured by qPCR. The relative expression was calculated to the level of *Porl2a*. Box-and-Whisker plot, horizontal bars indicate the median, boxes indicate 25^th^ to 75^th^ percentile, and the whiskers indicate 10^th^ and 90th percentile. Three independent experiments (n = 6). Cells were washed with PBS and epinephrine was measured by mass-spectrometry. Each dot represents and individual sample and the bar represents the mean ± SEM (n = 3). T lymphocytes in spleen and MLN of control and Th-CKO mice were characterized by flow. **(C)** Total TCRβ+ T cells, CD4+ T cells, CD8+ T cells, and FOXP3+ Treg cells were measured by flow cytometry. **(D)** T_H_17 cells were differentiated from naïve CD4+ T cells of control and Th-CKO mice and IL-17a-positive CD4+ T cells were quantified. Each dot representative of individual mouse and the bar represents the mean ± SEM (n = 2).

To investigate the role of epinephrine expression in T_H_17 cells during EAE induction, we generated a conditional knockout of *Th* in T_H_17 cells by breeding mice with *Th*-floxed to mice with ROR-γt-*Cre* (Th-CKO mice). We confirmed the expression of *Th* in SC-infiltrating T_H_17 cells from control mice; this was strongly decreased or undetectable in T_H_17 cells from Th-CKO mice (**Figure 5A**). There was comparable expression of other genes (*Ddc*, *Dbh*, and *Pnmt*) in T_H_17 cells between control and Th-CKO mice (data not shown). To determine whether the epinephrine level is decreased in T_H_17 cells from Th-CKO mice, T_H_17 cell differentiation was performed on naïve CD4+ T cells from either control mice or from Th-CKO mice. The epinephrine level was measured on day 3 of *in vitro* differentiation. The percentage of IL-17+ T_H_17 was 15–32% in both control and Th-CKO mice. There was a significant decrease in *Th* transcript and an approximately 50% decrease in epinephrine level from Th-deficient T_H_17 cells compared to control T_H_17 cells (**Figure 5B**).

Th-CKO mice are developmentally normal with a normal growth pattern compared to control mice. They also exhibit normal development of the immune system with comparable T cell subsets in peripheral lymphoid organs including the CD4:CD8 ratio and the frequency of Treg cells (**Figure 5C**), suggesting TH expression in T_H_17 cells is not critical for T cell development and differentiation. We also investigated whether TH expression or epinephrine production is required for proper T_H_17 differentiation *in vitro*, and equivalent numbers of T_H_17 cells were generated from CD4+ T cells from Th-deficient or from Th-sufficient mice (**Figure 5D**).

### Epinephrine Expression in T_H_17 Cells Does Not Alter EAE Development

**Figure 6:**
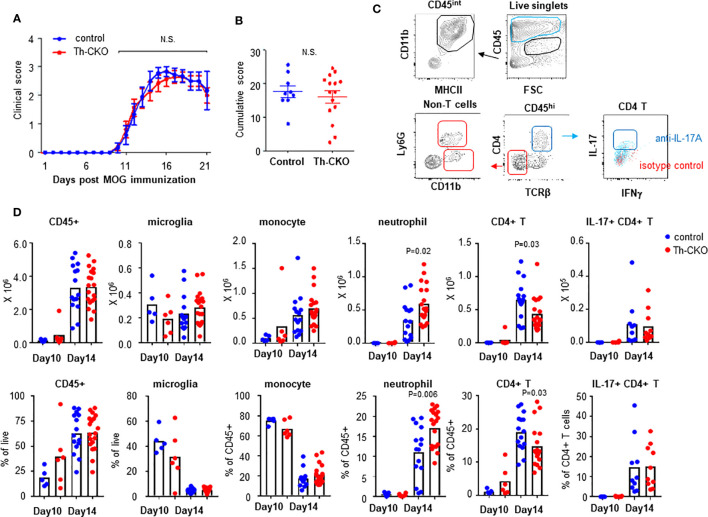
EAE induction and infiltration of leukocytes in CNS in control and Th-CKO mice. EAE was induced by immunization with MOG peptide in CFA followed by Pertussis toxin injection in 10-12 weeks old female control and Th-CKO mice and disease severity was daily determined throughout the time-course of disease development. Daily clinical score **(A)** and accumulative score from individual mouse **(B)** was calculated. Values represent mean ± SEM with n = 9-15 mice per group. Mice were sacrificed at day 10 or day 14 post immunization and spinal cord was harvested to investigate infiltrating leukocytes. **(C)** A representative image of flow images. **(D)** Number (upper row) and frequency (bottom row) of each leukocyte subset were calculated in spinal cords. Blur dot represents the cells from control mice and red dot represents the cells from Th-CKO mice. Each dot represents an individual mouse and the bar represents the mean (n = 3). N.S., not significant.

To test whether epinephrine production in T_H_17 cells plays a role *in vivo* in EAE, we decided to investigate the role of Th-sufficient wild type (control) and Th-deficient T_H_17 cells in the EAE mouse model. When control mice were immunized with MOG_35-55_ peptide in complete Freund’s adjuvant and pertussis toxin, we observed that the control mice developed a monophasic disease characterized by ascending paralysis 10–11 days after immunization. The disease incidence (control mice: 8/9 and Th-CKO mice: 12/15) and the kinetics of disease onset and progression was similar in both strains (**Figure 6A**). Both control and Th-CKO EAE mice developed comparable cumulative EAE scores (**Figure 6B**). These data suggest that epinephrine expression in T_H_17 cells does not affect the pathogenesis of EAE. Since epinephrine could be produced from the salvage pathway, an alternative explanation, that the residual epinephrine was enough or Th-CKO T_H_17 cells indeed produce sufficient epinephrine *in vivo* for local BBB breach, remain a possibility.

We compared subsets of helper T cells in peripheral lymphoid organs to confirm the immunization induced T_H_17 and T_H_1 differentiation. There was similar percent of each helper T cell subset including Tregs, T_H_17 and T_H_1 in spleen and LN from both control and Th-CKO mice (**Supplementary Figure 1**). These data suggest reduced epinephrine production in T_H_17 cells does not alter helper T cell differentiation.

We also confirmed infiltration of lymphocytes into the central nervous system (CNS) in myelin oligodendrocyte glycoprotein (MOG)-immunized control and Th-CKO mice. Mice were sacrificed at day 10 (early time point of disease) and 14 (peak time point of disease) post MOG-immunization. Total CD45+ cells and leukocyte subsets were examined in control and Th-CKO mice (**Figure 6C**). There was a similar percent and number of CD45^int^CD11b+MHCII+ microglia present in both strains (**Figure 6D**). As expected, infiltration of CD45^hi^ circulating leukocytes was observed at the early stage of disease (day 10) and this was significantly increased at day 14 (about from 20–40% to 60%). There was a similar percent of CD45^hi^ cells in control and Th-CKO mice (**Figure 6D**). The infiltration of monocytes (SSC^lo^Ly6G-CD11b^hi^MHCII^+^) was prominent at both early and peak disease time points, but not different between control and Th-CKO mice. Neutrophil (SSC^hi^CD11b^hi^Ly6G+) infiltration was great at the peak disease time point (~15% of CD45^hi^ cells) in both number and percentage. Since *Th* is selectively lost in T_H_17 cells, we compared the infiltration of T cells in CNS. Among the CD45^hi^ leukocytes, the percentage of CD4+ T cells was small (~ 5% of CD45^hi^) and not different between control and Th-CKO mice on day 10 post immunization (**Figure 6D**). Infiltration of CD4+ T cells was increased on day 14 (~20% of CD45^hi^). There were fewer CD4+ T cells in Th-CKO mice, but the frequency of IL-17+ CD4+ T cells remained similar in both groups (**Figure 6D**).

**Figure 7:**
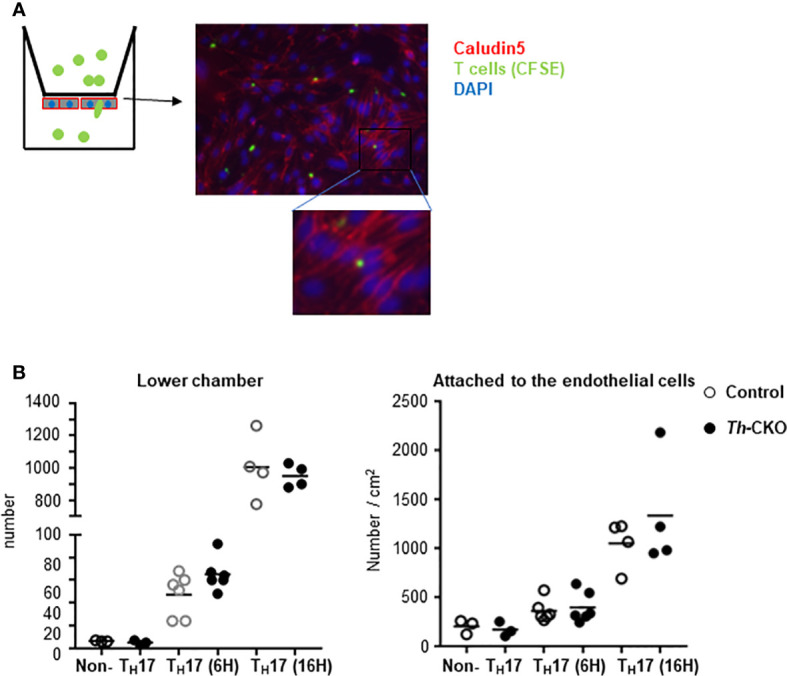
Transmigration of T_H_17 cells through brain endothelial cells. **(A)** Diagram of experiments and a representative image of T cells in the endothelial cells. Brain microvascular endothelial cells are plated on the bottom layer of transwell filter. When the cells are confluent, CFSE-labeled T cells were loaded in the upper chamber of a transwell apparatus. On day of assay, T_H_17 or non-T_H_17 cells were isolated from dLNs from day 10 post MOG peptide immunized mice. Transmigrated T cells in the lower chamber were counted and the endothelial cells and T cells were fixed and stained for Claudin. **(B)** Number of cells in the lower chamber (graph on the left) and number of cells in the endothelial cells was counted from 5 independent images per sample and normalized to the number per cm^2^ (graph on the right). Each dot represents an individual mouse and the bar represents mean (n = 2).

Because there was a trend toward fewer T cells in the spinal cords of Th-CKO mice, we decided to investigate transmigration of Th-deficient T_H_17 cells through a brain endothelial cell membrane. Primary mouse brain endothelial cells were cultured as a monolayer, and tight junction formation was confirmed by the expression of claudin and occludin (reviewed in (20)). Brain endothelial cells were coated on the bottom of the upper-chamber of a transwell apparatus and T_H_17 cells from either control mice or Th-CKO mice were loaded on the upper chamber for 6 or 16 h (**Figure 7A**). T_H_17 cells migrate through the endothelial cell layer starting at 6 h and the number of migrated cells was significantly increased at 16 h, however, there was no difference in migration of T_H_17 cells from control mice or Th-CKO mice (**Figure 7B**), demonstrating that diminished epinephrine expression does not impede penetration through the BBB.

## Discussion

Inflammation in the CNS is initiated by activated and antigen-specific lymphocytes. A specific chemokine and chemokine receptor (CCL20 and CCR6) are known to play a key role in leukocyte infiltration into the CNS, however, the mechanisms allowing migrated leukocytes to breach the tight junctions of the endothelial layer are not clearly identified. Epinephrine expression has been observed in lymphocytes, and increased epinephrine positively correlates with CNS inflammation, but the specificity and function of epinephrine in autoimmune disease is still being debated. In this study, we show that epinephrine production is induced in T_H_17 cells, but not in other helper CD4+ T cells. Epinephrine production by T_H_17 cells may be required for efficient infiltration of CD4+ T cells into brain parenchyma, but not required for T_H_17 differentiation or progression of EAE disease.

Multiple immune cell types are believed to be required for the pathogenesis of EAE, including T cells, monocytes and microglia. Numerous lines of evidence indicate that multiple sclerosis (MS) is driven by CD4 + T cells (21, 22), and EAE is a classic model of CD4+ T cell-mediated autoimmunity. Effector T cells, including T_H_1 and T_H_17 cells, are key pathologic cells for EAE pathogenesis. CD4+ T_H_1 cells produce high amounts of IFNγ, IL2 and TNFα, and the adoptive transfer of T_H_1 cells can induce EAE in recipient mice (23). Administration of IFNγ exacerbates MS. This has been attributed to the pathologic effect of IFNγ on MHC II expression in the CNS and the production of chemokines that attract macrophages and monocytes (24). The pathogenic function of T_H_17 cells in EAE was uncovered by cytokine-deficient models of EAE. Mice deficient in IL-12, IFNγ and TNFα (key cytokines for T_H_1 induction and function) develop severe EAE (25), but mice deficient in IL-23 (a key cytokine for T_H_17 differentiation) were completely protected from EAE (26). The ability of T_H_17 cells to produce proinflammatory cytokines, IL-17A/F, GM-CSF and CXCL8 (neutrophil attractant) suggests that T_H_17 cells can contribute to inflammation in the CNS (27–29). Sustained expression of IL7Ra was observed in human pathogenic T_H_17 cells (19), but whether signaling through IL7R/IL-7 in T_H_17 cells is necessary for their pathogenicity is controversial. IL7R signaling in the absence of IL-23 signaling did not induce T_H_17 cells to become pathogenic, rather it enhanced the pathogenic function of IFNγ-producing T_H_1 cells (30). IL-17 transcript is increased in chronic MS lesions (31), and neutralizing IL-17 activity shows partial amelioration in disease severity (32). IL-17A levels are elevated in the CSF of relapsing-remitting MS (RRMS) patients and a combination of IL-17A and IL-6 may disrupt the BBB by decreasing the expression of tight junction-associated genes (33). More recently, IL-17 was found to increase secretion of IL-1β from monocytes, which primes pathogenic T cells in early EAE (34).

Epinephrine and its pathway-related genes have been reported to be expressed in Treg cells and effector CD4+ T cells (15, 35), but we could not confirm the result in either human Tregs or in murine Tregs. We isolated functional Treg cells based on their low levels of CD49d and CD127 as well as CD25+ Tregs which was used as a marker of Tregs in the previous study. In humans, CD25 is a much less specific marker for suppressive FOXP3+ Treg cells than in mice (36). Indeed, a high percentage of T_H_17 CD4+ T cells are observed in PBMCs, and thus potentially contaminated the CD25+ Treg preparation. Regardless of the source of Tregs, both *TH* and *PNMT* expression was negligible. We detected expression of *PNMT*, but not *TH*, in circulating T_H_17 cells. The low level of *PNMT* in T_H_17 cells may reflect their origin in blood of healthy individuals. Analysis of gene expression and epinephrine production in infiltrating T_H_17 cells from MS patients could help address the significance of this pathway.

Our study clearly showed epinephrine expression in T_H_17 cells, and its production was decreased in Th-deficient T_H_17 cells *in vitro*. Although a decrease in epinephrine production was present in Th-deficient T_H_17 cells *in vitro*, transmigration through brain endothelial cells was not altered in Th-deficient T_H_17 cells compared to wild type T_H_17 cells. This is not due to altered T_H_17 differentiation in Th-deficiency since IL-17 production by T_H_17 cells was normal both *in vitro* and in dLNs after MOG-immunization. We also observed similar levels of IFNγ+/IL-17 double-positive cells in dLNs after MOG-immunization. These data suggest that Th-deficient T_H_17 cells can differentiate to pathogenic T_H_17 cells in an EAE model. Intact production of IL-17 from Th-deficient T_H_17 cells may be another mechanism of disrupting BBB and EAE development or the reduced epinephrine present in Th deficient mice may still be sufficient for induction of EAE. The lack of difference in T cell infiltration and disease progression between Th-CKO mice and control mice could be due to the model we used. PT is a potent inflammatory agent which can induce a BBB breaching; perhaps without its use, we may have seen a clearer difference in cell infiltration. Investigation of adoptive transfer of T_H_17 cells from Th-CKO mice and from control mice could also provide a valid answer to this question. Other potential explanation is that Th-deficient T_H_17 cells *in vivo* may have a secondary mechanism of epinephrine production by uptake of dopamine. Dopamine is synthesized in several tissues including the gastrointestinal system (37), and participate regulation of lymphocytes (38). We did not measure the dopamine production in Th-CKO mice, but comparable T_H_ development and T_H_17 differentiation in Th-CKO mice suggest normal production of dopamine in Th-CKO mice and this could be the source of epinephrine synthesis in Th-deficient T_H_17 cells. Nonetheless, the *in vitro* transmigration assay may exclude this possibility since there was no dopamine added during the assay.

Phagocytes are another important cell type during EAE progression (39, 40). They present antigen and secrete proinflammatory cytokines. There is a significant positive correlation between infiltrating monocytes and progression to the paralytic stage of EAE. Inhibition of infiltration of monocytes by chemokine receptor blockade can block or relieve the severity of EAE severity (41). However, it is not clear which cell type is critical for each stage of disease progression. Both T cell and myeloid cell infiltration was observed in EAE progression, but the relative frequency of each cell type was different. Infiltration of monocytes was prominent as early as day 10 post-immunization and maintained until a later stage of disease. However, T cell infiltration only exceeded monocytes infiltration at day 14. These data suggest that monocytes contribute more than T cells during the initiation of EAE, and T cells contribute more to EAE progression. We also found that neutrophil infiltration was greater in Th-CKO mice. Catecholamines (epinephrine and norepinephrine) can regulates neutrophil adhesion and demargination. Acute stimulation of human neutrophils with epinephrine reduced adhesion to endothelial cells by down-regulation of adhesion molecule expression, E-selectins (42). Epinephrine also can reduce trafficking of neutrophils or fMLP-induced migration, and these regulatory functions depend on signaling through β2-adrenoreceptor (43, 44). We do not know whether the increase in neutrophils in Th-CKO mice was actively induced by Th-deficient T_H_17 cells or merely compensatory. This could reflect altered chemokine production by Th-deficient T_H_17 cells and needs to be investigated.

Taken together, it can be concluded that epinephrine production and the enzymes which are required for *de novo* synthesis of epinephrine are induced in T_H_17 cells. T_H_17-specific knockdown of Th can decrease epinephrine production. Although an infiltration of CD4+ T cells into CNS was decreased, disease progression and BBB breach were not affected by decreased epinephrine production from T_H_17 cells.

## Data Availability Statement

The raw data supporting the conclusions of this article will be made available by the authors, without undue reservation.

## Ethics Statement

The animal study was reviewed and approved by the Committee on the Ethics of Animal Welfare of the FIMR. Written informed consent was obtained from the owners for the participation of their animals in this study.

## Author Contributions

PY, SK, HT, and GH performed the experiments. PC and HI generated and provided conditional knockout mice. YZ supervised and interpreted the *in vivo* experiments. SK and BD designed the study, supervised the experiments, interpreted the data, and prepared the manuscript. All authors contributed to the article and approved the submitted version.

## Funding

This study was supported by NIAMS, US National Institute for Health (R01 AR065209 to SK and BD).

## Conflict of Interest

The authors declare that the research was conducted in the absence of any commercial or financial relationships that could be construed as a potential conflict of interest.
