# Supplementary material for: Epinephrine Production in Th17 Cells and Experimental Autoimmune Encephalitis
Source: Front Immunol. 2021 Feb 22;12:616583. doi: 10.3389/fimmu.2021.616583 (PMC7937652; doi:10.3389/fimmu.2021.616583)
Supplement: Supplementary file 1 [file DataSheet_1.docx]

**Supplement figure 1**: T_H_ differentiation in Th-CKO mice by MOG-immunization. Both wild type and Th-CKO mice were immunized with MOG peptide in CFA s.c. Mice were sacrificed at day 10 and day 14 post immunization and compared T_H_1, T_H_17 and Treg cells in dLNs by IFNγ, IL-17A and FOXP3, respectively. **(A)** a representative flow data of T cells. Frequency of each T_H_ cells in **(B)** and **(C)**. Each dot represents an individual mouse and the bar represents the mean ± SEM (n=2 for day 10 and n=4 for day 14).
